# Regional determinants of quality of care for sick children: A multilevel analysis in four countries

**DOI:** 10.7189/jogh.14.04053

**Published:** 2024-03-15

**Authors:** Hwa-Young Lee, Jan E Cooper, Margaret E Kruk

**Affiliations:** 1 Graduate School of Public Health and Healthcare Management, The Catholic University of Korea, Seoul, Republic of Korea; 2 Catholic Institute for Public Health and Healthcare Management, The Catholic University of Korea, Seoul, Republic of Korea; 3 Department of Global Health and Population, Harvard T.H. Chan School of Public Health, Boston, Massachusetts, USA

## Abstract

**Background:**

The limited impact of increased access to care on improvements in health outcomes during the Millennium Development Goal era has been attributed, in part, to inadequate quality of care. We identified regional factors associated with health service quality for sick child care in low-income countries and examined whether provider factors interact with regional factors to affect the quality of care.

**Methods:**

We conducted cross-sectional random intercept four-level linear regression using the most recent Service Provision Assessment and Demographic Health Survey data from four countries (467 from the Democratic Republic of Congo 2018, 2425 from Afghanistan 2018, 2072 from Nepal 2015, and 813 from Senegal 2017). The outcome variable was the service quality score for sick child care, which was measured as the percentage of clinically recommended tasks completed in the integrated management of childhood illness (ranging from 0 to 100). Regional factors were selected based on the High-Quality Health System Framework.

**Results:**

The service quality score was found to be positively associated with the proportion of large facilities (β = 8.61; *P* = 0.004) and the proportion of providers ranked in the top fifth for service quality score (β = 30.15; *P* < 0.001) in the region. We identified significant cross-level interactions between provider qualifications (β = −16.6; *P* < 0.001) or job descriptions (β = 12.01; *P* = 0.002) and the proportion of providers in the top fifth for service quality scores within the region. As the proportion of top-performing providers in a region increased, the increase in the service quality score was more pronounced among providers who were nonmedical doctors or did not have job descriptions than among their counterparts.

**Conclusions:**

Our findings indicate that the quality of care for sick children in a region improves with a greater proportion of high-performing providers or larger facilities. Providers who are not medical doctors, or those who have specific job descriptions, tend to benefit more from the presence of these top-performing providers.

During the Millennium Development Goal era, the primary reason for poor population health in low- and middle-income countries (LMICs) was identified as the lack of timely access to health care. In response to this issue, a significant portion of global efforts has focussed on providing infrastructure, such as building health facilities and supporting equipment and medication, as well as deploying health professionals to underserved areas. However, recent evidence indicates that in LMICs, simply expanding access to health care has not led to improved health outcomes, which has partly been attributed to the substandard quality of care provided [1–3].

A growing body of evidence has shown that the quality of health services varies widely across different areas [4–6]. Health service delivery systems are complex and adaptive; they operate through interactions of multiple factors at various levels and adapt to shifts in economics, policy priorities, and public health threats [7–9]. A better understanding of how the attributes of multiple players at multiple levels influence the quality of the health system would be the first step in elucidating the pathways for fundamentally improving health services.

Numerous studies have explored the factors influencing the utilisation of health services [10,11]. However, relatively few studies have investigated the determinants of health system quality or the services provided. Most of these studies have concentrated on micro-level factors, such as the characteristics of patients and providers [12–15]. Research on the area-level determinants of care quality is limited and has primarily focussed on socioeconomic traits [5,6]. To date, there has been no comprehensive investigation into the regional-level determinants of health service quality, nor has there been an exploration of the interaction between area-level factors and provider characteristics using a multilevel approach.

Thus, we aimed to address two primary questions: What are the region-level characteristics associated with health service quality for sick child care? How do provider characteristics interact with region-level factors to influence the quality of care? To investigate these issues, we developed a conceptual framework positing that the quality of health services for sick child care is influenced by various factors across different levels (**Figure 1****)**. The hypothesised pathways outlined in this framework are detailed in Appendix S1 in the **Online Supplementary Document**.

**Figure 1:**
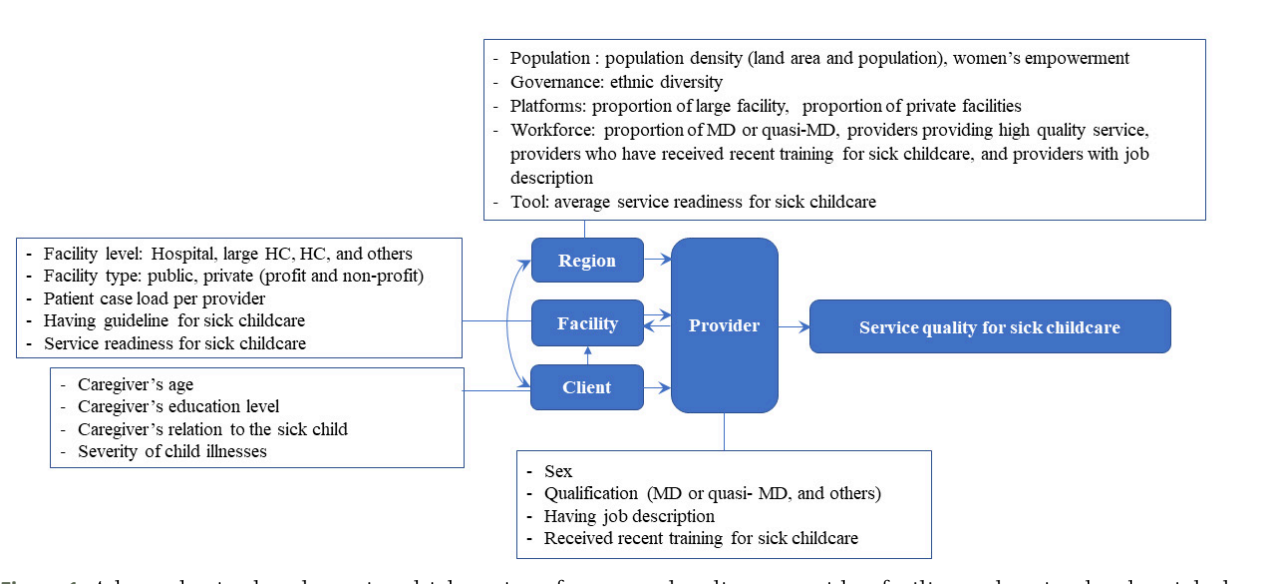
A hypothesised pathway in which various factors at the client, provider, facility and region levels might be associated with the quality of health services for sick child care (constructed by the authors).

## METHODS

### Study design and data source

We conducted a multicountry, cross-sectional observational analysis using combined data sets from two distinct sources. The primary data set originated from the Service Provision Assessment (SPA) surveys, which were carried out by the Demographic and Health Survey (DHS) programme in partnership with national statistics agencies in the surveyed countries (see details in Appendix S2 in the **Online Supplementary Document**). The SPA surveys aim to evaluate the capacity of health systems in LMICs. Nationally representative samples of health facilities were selected from the national master facility list, which includes all public and nonpublic health facilities in the formal sector, from primary to tertiary care levels. We initially identified countries where care observations and exit interviews for sick child care in SPAs were conducted after 2015. This list comprised the Democratic Republic of Congo (2017–18), Haiti (2017–18), Senegal (2018–19), Afghanistan (2018–19), and Ethiopia (2021–22), hereafter referred to by the final year of each survey. We excluded Haiti due to the absence of information on ethnicity in the DHS, which is an important regional characteristic for our study. We also excluded Ethiopia because its survey was conducted during the coronavirus disease 2019 (COVID-19) pandemic, a period characterised by significant disruptions in the health system that likely affected service delivery and provider behaviour, potentially compromising data comparability [16]. For Senegal, where data were available for two consecutive years, we included only the most recent data set, thereby excluding the data from 2018. Consequently, the countries included in our study were Afghanistan (2019), Nepal (2015), the Democratic Republic of the Congo (2018), and Senegal (2019).

We extracted data on women’s empowerment level and ethnic diversity, which are region-level characteristic variables, from the DHS. The DHS data selected for each country were those completed closest to, but prior to, the year of the SPA: 2015 for Afghanistan, 2011 for Nepal, 2014 for the Democratic Republic of the Congo, and 2018 for Senegal. Both the SPA and DHS are designed to be representative at the regional level. However, the Afghanistan SPA was an exception, as it targeted only urban areas in seven major provinces out of all 34 provinces in the country.

### Outcome variables

The outcome variable was a technical quality score for sick child care, calculated as the percentage of tasks completed during consultations relative to those clinically recommended across four domains outlined in the Integrated Management of Childhood Illness (IMCI) guidelines: patient history taking, physical examination, testing/management, and client education and counselling, with a possible range of 0 to 100 (Table S1 in the **Online Supplementary Document**) [17]. We assigned all tasks equal weights. The utility of this indicator has been well-established in previous studies [18–22].

### Child and caregiver, provider, and facility characteristics

For caregiver characteristics, we included the age of the client (caregiver of a sick child), categorised as under 25, 25–40, and over 40 years; their education level (less than secondary graduate and secondary graduate or higher); and their relationship to the sick child (mother, father, or other). The severity of the child's symptoms was assessed by summing the reasons the caregiver brought the child to the facility, with a possible range of 0 to 8. Provider characteristics included sex (male or female); qualifications (medical doctor (MD), quasi-MD (defined as an advanced practice clinician (APC) or paramedical clinical officer (PCO)), and nurses/midwives and aides/assistants); whether they had a job description (yes or no); and whether they had received training for sick child care in the past two years (yes or no). Facility characteristics included the level of the facility (hospital, large health centres, health centres, health posts/dispensaries, etc.); management authority (public, private for-profit, and private not-for-profit); the sick child patient load per provider per facility on the survey day (two or fewer, more than two but three or fewer, and more than three); whether IMCI guidelines for the care of sick children were observed (yes or no); and the service readiness score for sick child care (ranging from 0 to 1). The service readiness score was developed to measure structural quality according to the World Health Organization (WHO) Service Availability and Readiness Assessment guidelines [23]. This score was calculated as the proportion of all items related to basic equipment and essential medicines for sick child care. We determined the sick child patient load by dividing the number of sick child clients on the day of the SPA visit by the number of providers attending to sick children on the same day.

### Regional characteristics

Regional factors were chosen based on the High-Quality Health System Framework [1]. We selected the highest political unit within each country as the region level, taking into account data availability and the authority of these units to make health policy decisions. Detailed information about the regions in each country is provided in Table S2 in the **Online Supplementary Document**. While the official term for the highest political unit varies by country, we will refer to it as ‘region’ in the subsequent text.

We extracted information on population density (1000 population per km^2^) from different sources for each country (Table S3 in the **Online Supplementary Document**) [24–28]. If data on population density for the same year as the SPA survey year were unavailable, we used information from the closest available year. We assessed women's empowerment using DHS data, following the approach of Lewis et al. [15], which included 25 indicators of empowerment that are most likely to be associated with seeking and obtaining high-quality care for a child. An individual score for women's empowerment was calculated based on the proportion of these 25 empowerment factors experienced by a woman. Subsequently, we computed the proportion of women in each region who fell into the top quintile (5th) for women's empowerment scores across all four countries (0–1). We quantified region-level ethnic diversity using Simpson's Index of Diversity, also derived from DHS data (0–1), with higher scores indicating greater diversity [29]. We constructed other region-level variables using corresponding individual-level variables from the SPA data set (0–1); these included the proportion of large facilities (hospitals and large health centres); private facilities; MDs/quasi-MDs; providers in the top quintile for technical quality scores in the care of sick children across all four countries; providers with recent IMCI training; providers with job descriptions; and the average service readiness score for sick child care in each region.

### Statistical analysis

We performed complete-case analyses after removing missing data. First, we described the characteristics of the final analytic sample, both overall and by country. Subsequently, we explored the bivariate associations between each independent variable and the technical quality of sick childcare. For the main analyses, we utilised a four-level random-intercept linear regression model, in which level 1 represents a sick child, which is nested within level 2 (the provider), which in turn is nested within level 3 (the facility), which is finally nested within level 4 (the region).



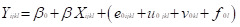




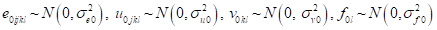


*Y_ijkl_* denotes the technical quality score for the service that individual child *i* received from provider *j* during the visit to facility *k* located in region *l*. β_0_ represents the average technical quality score of all individual children across all countries, and *e_0ijkl_*, *u_0jkl_*, *v_0kl_*, and *f_0l_* represent the random effects (i.e. residuals) associated with children, providers, facilities, and regions, respectively. The residuals were assumed to be identically and independently distributed at each level. Five distinct model specifications were estimated according to the general modelling structure outlined above: a null model and models M1 through M5. The null model included only an intercept term in the fixed part of the model. Subsequent models, M1 to M5, progressively incorporated additional variables: client characteristics in M1, provider characteristics in M2, facility characteristics in M3, regional characteristics in M4, and a country variable in M5. The sequential addition of variables allows for an assessment of the incremental contribution of each set of variables to the model. The validity of the final model was assessed by verifying the approximate normal distribution of residuals. We created a histogram and calculated skewness and excess kurtosis, which is obtained by subtracting 3 from the proper kurtosis of the residuals at each level [30]. Although there are no official rules about cut-off criteria to decide how large skewness or kurtosis values must be to indicate nonnormality, skewness and excess kurtosis at all levels were included between −1 and +1, which Mishra et al. [30] suggest as indicative of approximate normality (Figure S1 in the **Online Supplementary Document**). Statistical significance was determined using a two-sided *P*-value, with a threshold set at less than 0.05.

We calculated variance partitioning coefficients (VPCs) to examine the proportion of the total variance attributed to each level, computed as:



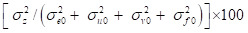


Finally, we included the interaction terms of provider and region characteristics (M4-1 ~ M4-8). All analyses were performed using Stata, version 17 (StataCorp LLC, College Station, TX, USA)

## RESULTS

### Descriptive statistics

After excluding missing data, we identified a total of 5777 sick children who received care from 2407 providers at 1913 facilities across four countries (**Table 1**). Overall, 9.2% of children, 5.6% of providers, and 4.4% of facilities had missing values for any of the variables included, with the highest percentage of missing data reported in Afghanistan. A comparison of the characteristics between the original sample (n = 6363) and the final analytic sample (n = 5777) by country (Table S4–5 in the **Online Supplementary Document**) showed that the differences in percentages across each category were within a 2–3% range.

**Table 1:** Descriptive statistics of the final analytic sample

	n (%)	Quality score, x̄ (SD)	Minimum ~ maximum
**Client level**	5777 (100)	31.5 (13.7)	0 ~ 86.4
Caregiver’s age (years)			
*>10 and ≤25*	2442 (42.3)	31.2 (13.9)	
*25–40*	2861 (49.5)	31.9 (13.6)	
*>40*	474 (8.2)	30.1 (13.0)	
Caregiver’s education level			
*Lower than secondary graduate*	2817 (48.8)	31.6 (14.1	
*Secondary graduate and above*	2960 (51.2)	31.3 (13.3)	
Relation to child			
*Mother*	4607 (79.7)	31.9 (13.7)	
*Father*	667 (11.5)	29.8 (13.2)	
*Others*	503 (8.7)	30.0 (14.9)	
Total numbers of the child’s symptoms			
*0*	69 (1.2)	27.2 (14.5)	
*1*	872 (15.1)	26.7 (14.1)	
*2*	1665 (28.8)	29.8 (13.8)	
*3*	1448 (25.1)	32.5 (12.9)	
*4*	985 (17.1)	34.1 (12.8)	
*5*	512 (8.9)	35.4 (13.5)	
*6*	176 (3.1)	36.9 (13.1)	
*7*	42 (0.7)	35.0 (12.3)	
*8*	8 (0.1)	36.9 (15.6)	
**Provider level**	2407 (100)	32.1 (13.7)	0 ~ 81.8
Gender			
*Male*	1897 (78.8)	32.2 (13.9)	
*Female*	510 (21.2)	31.8 (13.1)	
Qualification			
*MD*	981 (40.8)	31.7 (13.0)	
*quasi-MD and others*	1426 (59.2)	32.4 (14.2)	
Having job description			
*No*	1388 (57.7)	30.8 (13.2)	
*Yes*	1019 (42.3)	33.9 (14.2)	
Recent training for sick childcare			
*No*	1931 (80.2)	34.1 (13.7)	
*Yes*	476 (19.8)	35.1 (13.4)	
**Facility characteristics**	1913 (100)	32.2 (13.8)	0 ~ 81.8.
Facility level			
*Hospital*	685 (35.8)	32.0 (13.1.)	
*Large health centre*	158 (8.3)	35.7 (12.7)	
*Health centre*	778 (40.7)	32.8 (13.8)	
*Health post and others*	292 (15.3)	29.1 (15.4)	
Facility type			
Public	1397 (73.0)	32.1 (14.2)	
Private for profit	210 (11.0)	31.4 (13.2)	
Private not for profit	306 (16.0)	33.4 (12.2)	
Patient load per day per provider			
*≤2*	1590 (83.1)	32.7 (13.7)	
*>2 and ≤3*	133 (7.0)	30.2 (15.6)	
*>3*	190 (9.9)	29.3 (13.2)	
Guideline of IMCI observed			
*No*	898 (46.9)	30.5 (13.6)	
*Yes*	1015 (53.1)	33.7 (13.8)	
Service readiness score for sick childcare		0.64 (0.08)	0.46 ~ 0.81
**Regional level**	52 (100)		
Proportion of women in top 5th in empowerment score		0.18 (0.13)	0.01 ~ 0.49
Population density (1000 per km^2^)		0.25 (0.90)	0.01 ~ 6.46
Ethnic diversity score		0.41 (0.28)	0.01 ~ 0.84
Proportion of large facilities (hospital and large health centre)		0.19 (0.20)	0.00 ~ 0.93
Proportion of private facility		0.31 (0.30)	0.00 ~ 0.95
Proportion of MD or quasi-MD		0.27 (0.34)	0.00 ~ 1.00
Proportion of providers in the top 5th quintile in quality score		0.22 (0.19)	0.00 ~ 0.90
Proportion of providers who received recent IMCI training		0.24 (0.18)	0.00 ~ 0.88
Proportion of providers with job description		0.50 (0.27)	0.29 ~ 1.00
Average service readiness score for sick childcare		0.66 (0.08)	0.43 ~ 0.80

IMCI – Integrated Management of Childhood Illness, MD – medical doctor, SD – standard deviation, x̄ – mean

The sample characteristics differed across countries (Table S6 in the **Online Supplementary Document**). For instance, the percentage of caregivers who had completed secondary education varied widely, from 20.9% in Senegal to 58.6% in Nepal. In Afghanistan, all health care providers had either an MD or a quasi-MD qualification, while in Senegal, only 8.8% held such qualifications. Hospitals accounted for 71.2% of health care facilities in Afghanistan, but represented only 7.6% of them in Senegal. Moreover, in Senegal, public facilities made up more than 90% of the total number of health facilities, in contrast to Afghanistan, where only 17.8% of facilities were public.

### Factors associated with quality of service for sick child care

The majority of characteristics pertaining to the child, caregiver, provider, facility, and region were associated with the quality of service for sick child care, as indicated by bivariate analyses (Table S7 in the **Online Supplementary Document**). According to a fully adjusted four-level multivariate regression analysis, children cared for by fathers were more likely to receive a lower quality of care than those cared for by mothers (β = −1.03; *P* = 0.024) (**Table 2**). More severe symptoms in a child were linked to a higher quality of service (β = 1.41; *P* < 0.001). Providers with a job description and those who had undergone IMCI training in the past two years were more likely to deliver higher quality care for sick children than their counterparts (β = 1.32; *P* = 0.008 and β = 1.75; *P* = 0.003, respectively). Hospitals did not provide better quality service than lower-level facilities. Private for-profit facilities and those following IMCI guidelines were more likely to offer higher quality care than public facilities and those not following IMCI guidelines, respectively (β = 2.30; *P* = 0.016 and β = 2.12; *P* < 0.001, respectively). Facilities where providers saw more than three sick children per day had a quality score that was 2.58 points lower than that of the reference facilities where providers saw fewer than two sick children per day.

**Table 2:** Association between client-, provider-, facility- and region-level characteristics and quality score of sick child care from four-level linear regression

	Model 1		Model 2		Model 3		Model 4		Model 5	
	**β**		**β**		**β**		**β**		**β**	
**Client characteristics**										
Age (ref: ≤25)										
*25 − 40*	0.05	0.86	0.04	0.899	0.03	0.909	0.05	0.869	0.03	0.926
*>40*	−0.75	0.211	−0.74	0.22	−0.74	0.219	−0.67	0.266	−0.69	0.249
Education level (ref: Lower than secondary graduate)										
*Secondary graduate or above*	0.30	0.341	0.33	0.304	0.33	0.307	0.35	0.278	0.40	0.212
Relation to child (ref: Mother)										
*Father*	−1.13	0.014	−1.13	0.014	−1.09	0.018	−1.03	0.025	−1.03	0.024
*Sibling/grandparent/other*	−0.60	0.283	−0.59	0.292	−0.62	0.269	−0.63	0.262	−0.66	0.238
Total number of the child’s symptoms	1.41	<0.001	1.42	<0.001	1.42	<0.001	1.41	<0.001	1.41	<0.001
**Provider characteristics**										
Gender (ref: Male)										
*Female*			−0.08	0.882	0.01	0.992	0.13	0.813	−0.02	0.975
Qualification (ref: Non-MD)										
*MD*			−0.13	0.801	0.35	0.6	0.48	0.469	0.48	0.468
Having job description (ref: No)										
*Yes*			1.47	0.003	1.52	0.002	1.34	0.007	1.32	0.008
Having received recent IMCI training (ref: No)										
*Yes*			2.07	<0.001	1.74	0.003	1.76	0.003	1.75	0.003
**Facility characteristics**										
Facility level (ref: Hospital)										
*Large health centre*					1.19	0.229	1.23	0.207	0.97	0.321
*Health centre*					0.06	0.935	0.14	0.847	−0.07	0.917
*Health post and others*					0.66	0.529	0.45	0.658	1.00	0.339
Facility type (ref: Public)										
*Private for profit*					1.91	0.028	2.09	0.016	2.30	0.008
*Private not for profit*					−0.63	0.389	−0.39	0.589	−0.46	0.524
Patient case load (ref: ≤2)										
*>2 and ≤3*					−1.46	0.102	−1.45	0.102	−1.48	0.096
*>3*					−2.53	0.002	−2.58	0.001	−2.53	0.002
Guideline for IMCI observed (ref: No)										
*Yes*					2.27	<0.001	2.16	<0.001	2.12	<0.001
Service readiness for sick childcare					11.35	0.24	3.32	0.566	2.93	0.634
**Region characteristics**										
Proportion of top 5th quintile in women empowerment							−4.50	0.128	3.27	0.507
Population density (1000 per km^2^)							0.00	0.832	−0.38	0.369
Ethnic diversity							−0.93	0.531	−0.22	0.9
Proportion of large facilities							8.12	0.004	8.61	0.003
Proportion of private facilities							−1.41	0.461	−2.01	0.364
Proportion of MD or quasi-MD							−2.29	0.239	−2.44	0.305
Proportion of providers in top 5th in quality score							30.15	<0.001	29.68	<0.001
Proportion of providers who received recent IMCI training							0.26	0.915	−3.14	0.224
Proportion of providers with job description							−2.00	0.197	−2.69	0.101
Service readiness score for sick childcare							4.48	0.389	−3.05	0.711
**Country** (ref: Democratic Republic of Congo, 2018)										
*Afghanistan, 2018*									1.20	0.736
*Nepal, 2015*									−2.13	0.229
*Senegal, 2017*									3.25	0.14

IMCI *–* Integrated Management of Childhood Illness, MD – medical doctor, ref – reference

Children who received services at facilities in regions with a high concentration of large facilities and a greater proportion of providers ranked in the top fifth for service quality were more likely to receive higher-quality care. None of the country dummy variables, which represented the national context, were significant (**Table 2**).

### Cross-level interaction between provider and region characteristics

We generated cross-level interaction terms by combining all provider characteristics and regional characteristics that demonstrated a significant association with the service quality score; these factors were alternately included in the model (Table S8 in the **Online Supplementary Document**). Since none of the country variables in Model 5 reached significance (**Table 2**), we opted not to incorporate them into the interaction model.

**Figure 2:**
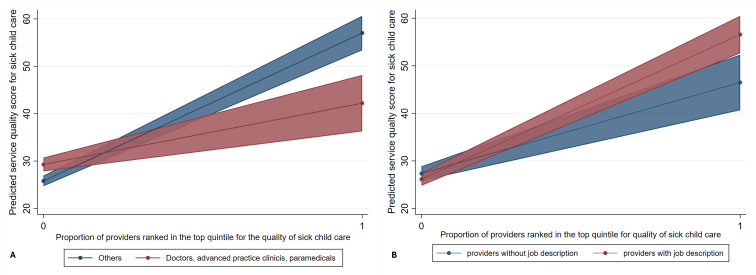
**Panel A.** Predicted service quality score for sick child care by MD (or quasi-MD) providers and those with other qualifications by the proportion of providers ranked in the top quintile for the quality of sick child care in the region. **Panel B.** Predicted service quality score for sick child care by providers with and without job descriptions by the proportion of the providers ranked in the top quintile for the quality of sick child care in the region.

We found that provider qualifications and job descriptions interacted with the proportion of providers in the top quintile of service quality scores within a region, influencing the quality of service received by individual sick children (β = −16.86; *P* < 0.001 and β = 12.01; *P* = 0.002, respectively). The negative coefficient for the interaction term between being an MD or quasi-MD and the proportion of top-quintile providers indicated that as the proportion of high-performing providers in a region increased, the predicted service quality score increased more significantly for non-MD providers (such as nurses, midwives, or aides/assistants) than for MDs or quasi-MDs (as shown in Model 4–2 in Table S8 in the **Online Supplementary Document**) (**Figure 2**, Panel A). Furthermore, the positive coefficient for the interaction term between having a job description and the proportion of top-quintile providers suggests that as the proportion of top-performing providers in a region increased, the predicted service quality score improved more for providers with a job description than for those without one. The proportion of large facilities in a region did not interact with any provider characteristics (as indicated in Models 4–5 and 4–8 in Table S8 in the **Online Supplementary Document**).

## DISCUSSION

The quality of clinical care provided during a sick child visit is influenced by a multitude of factors functioning at different levels within the health system, encompassing aspects related to the client, provider, facility, and region. To the best of our knowledge, this study is the first to explore the impact of area-level factors on the quality of health services for sick child care in LMICs. It also examined the interplay of characteristics at different levels that contribute to the delivery of high-quality child care. Our analysis revealed several significant findings.

First, we identified a significant cross-level interaction between the provider's qualifications and the proportion of providers ranked in the top quintile for the quality of sick child care within their respective regions. The disparity in service quality offered by non-MD providers across two distinct regional contexts – one where no providers were in the top quintile for the quality of sick child care, and another where all providers were classified in this category – was more pronounced than the difference in service quality observed among MD or quasi-MD providers between these same regions (**Figure 2**, Panel A).

The health care sector exemplifies a dynamic work environment that is constantly evolving with the advent of new technologies, innovative treatment options, or changing disease patterns [31]. Consequently, professional development and workplace learning actively occur both formally and informally [32]. We hypothesise that regions with a higher concentration of top-performing providers experience more active knowledge sharing and dissemination of best practices [33], which could enhance the overall quality of care for sick children. Such regions may not only foster a culture of excellence and commitment to high standards of care but also provide better access to resources, training, and support systems that facilitate formal and informal learning [34].

Our findings suggest that non-MD providers (such as nurses, midwives, aides, or assistants) who may initially have a broader range of skills and knowledge bases than MDs could also have greater potential for improvement. Consequently, they might benefit more significantly from learning opportunities or collaboration with high-performing peers [35,36]. In addition, the international focus on professional training has increasingly centred on nurses and midwives in addressing the health workforce crisis in resource-limited settings. The significance of providing these professionals with opportunities to transition to physician roles has been highlighted in previous studies [37,38]. Research indicates that, for many services, nonphysician clinicians (including nurses and midwives who are properly trained) can deliver care of a quality comparable to that of physicians, but at a lower cost [39,40]. Empirical evidence from Pakistan, Myanmar, and Sri Lanka demonstrates that the cost of training 2.5 to 3 nurses is equivalent to the cost of training one physician [41]. While peer learning has been gaining recognition as a valuable method for fostering ongoing professional development among health professionals [34], these dynamics may offer non-MD providers expanded opportunities for professional growth, particularly through peer learning.

Second, the provision of job descriptions for health care providers was not only positively associated with improved service quality in the care of sick children (M5 in **Table 2**) but also reinforced the association with regional factors (M4–3 in Table S8 in the **Online Supplementary Document**). Significantly, as the proportion of top-performing providers in a region increased, the quality of services provided by those with a defined job description improved more substantially than that provided by providers without a job description (**Figure 2**, Panel B).

The effectiveness of job descriptions in enhancing the motivation of health workers at various levels of care has been demonstrated in several earlier studies [42–44]. The WHO has recognised job descriptions as one of the tools that positively influences personnel performance [45]. Job descriptions keep providers aware of the objectives and responsibilities associated with their roles. This promotes clarity and a sense of professionalism [45,46], which in turn motivates them to perform to the best of their abilities. Our analysis suggests that the positive impacts of job descriptions may further enhance the effect of peer learning. Policymakers may wish to consider the extensive use of job descriptions, as they are easy to implement, relatively inexpensive, and can have an immediate impact [45].

Third, facility characteristics operated differently at the facility level and at the regional level in relation to the service quality for the care of sick children. While the quality of care provided at hospital-level facilities was comparable to that at lower-level facilities, a greater proportion of large facilities (hospitals and large health centres) within a region was positively associated with the quality of service in that region. Conversely, although individual private-for-profit facilities tended to offer better care than public facilities, the proportion of private-for-profit facilities in a region was not associated with a higher quality of care for sick children. The lack of quality difference between large and lower-level facilities suggests that the positive relationship between the proportion of large facilities in a region and the quality of service is not due to a compositional effect but rather a contextual effect. For example, large facilities might enhance care quality in the region by facilitating knowledge and experience sharing through organised activities or events. Health system networks that connect hospitals with lower-level facilities are increasingly being used as an approach to improve care quality via efficient care coordination and the sharing of knowledge, information, education, and learning activities [47]. In contrast, the lack of a similar positive effect from a greater proportion of private-for-profit facilities suggests that these facilities do not contribute to regional service quality through positive spillover effects, despite providing higher quality care than public facilities.

### Strengths and limitations

Our study has limitations. First, we constructed the quality indicator by identifying items recommended in evidence-based guidelines and matching them with items available in the SPA survey. Consequently, our quality indicator may not fully capture the entire spectrum of sick childcare quality. Nevertheless, the indicator was derived from a broad array of service items, as outlined in Table S1 in the **Online Supplementary Document**. Moreover, scores were broadly distributed across individuals and regions, suggesting the utility of the indicator. Notably, the same approach for constructing quality indicators has been employed in previous studies [20,48,49]. Therefore, we argue that this limitation does not undermine the validity of our findings. Second, the missing data rate was relatively high at 18.9% in the Afghanistan data set. However, when comparing the sociodemographic characteristics with those of the original sample, the differences were marginal, within 2–3%. Thus, it is unlikely that the missing observations introduced any significant bias. Third, although direct observation is considered the gold standard, this method is prone to observation biases and the Hawthorne effect, where providers may change their behaviour because they know they are being observed. Nonetheless, this would not result in significant bias, and any overall inflation of the quality score should occur uniformly unless this effect is correlated with the characteristics of the client, provider, or facility. Fourth, since our analysis was limited to only four countries, the findings may not be fully generalisable to all other LMIC settings. Fifth, provider qualifications are not entirely consistent across the countries included in our study. For instance, it is unclear whether the qualifications of an advanced practice clinician, known as an assistant medical officer (AMO) in Nepal, and a paramedical clinical officer (PCO) in Senegal, are equivalent. However, this potential inconsistency is unlikely to introduce bias because we aggregate MDs and all quasi-MD qualifications into a single category. Finally, the cross-sectional nature of our data set prevents us from establishing causal relationships between the factors examined and the quality of care provided for sick children. Despite these limitations, our study has several strengths. First, this inaugural study explored regional factors that influence the quality of health services for sick child care. Second, the quality of service in the SPA data set used for the current study was measured by direct observation, which is the most reliable method for assessing quality. Direct observation has been conclusively shown to be superior to routine indicators or record reviews [50].

## CONCLUSION

We identified two regional factors associated with the quality of sick child care that individual children receive: the proportion of top-performing providers and the proportion of large facilities in the region. These findings suggest that large facilities and top-performing providers may have a positive spillover effect on the quality of sick child care in the region. Additionally, we found that non-MD providers and providers with a defined job description might experience a more positive influence from top-performing providers. While future research to explore the underlying mechanisms would be valuable, our findings offer policymakers novel insights into optimising regional environments and provider mixes to improve the quality of care for sick children. Local governments, organisations, and community leaders are encouraged to create an ecosystem that maximises the positive influence of top performers and large facilities. This could involve the formation of a regional health care network where large facilities and top-performing providers play a central role in facilitating expertise sharing. Furthermore, programs that offer recognition or incentives to top performers who take on leadership roles in mentoring or training other health professionals are recommended. However, to design more effective programs, further investigation is necessary to fully understand the dynamics at play.

## Additional material


Online Supplementary Document
