# Supplementary material for: Regional determinants of quality of care for sick children: A multilevel analysis in four countries
Source: J Glob Health. 2024 Mar 15;14:04053. doi: 10.7189/jogh.14.04053 (PMC10939116; doi:10.7189/jogh.14.04053)
Supplement: Online Supplementary Document [file jogh-14-04053-s001.pdf]

eAppendix1. Hypothesized pathways by which factors at different levels may affect the service quality for sick child care

Since health services are delivered by providers, provider characteristics such as provider's sex, qualification, having job description or updated training will directly influence the quality of health service they offer (Barber, Bertozzi, & Gertler, 2007; Hansen et al., 2008; Leslie, Gage, Nsona, Hirschhorn, & Kruk, 2016; Sato et al., 2017; Soeters, Habineza, & Peerenboom, 2006). They also may indirectly affect service quality by influencing the characteristics of the facility where they work, for example by demanding that the facility introduce specific procedures or rules of operation. Although children are direct beneficiaries of health services, caregivers are the ones who make decision on utilization of services and assess the effectiveness (Pariyo, Gouws, Bryce, & Burnham, 2005). They may complain to provider or facility about the quality of the service they received, and ask for better care for their sick child. They can also have an impact on the health service quality by urging the local government to invest more in health service or by exercising their right to vote. We included a caregiver's age, education level, relation to the sick child and the severity of child illnesses as factors related to this. Health service quality depends on the provider incentives inherent to facility type or level and governance of a facility.

Region characteristics might affect the service quality from a macro perspective. We constructed the region-level variables based on the five sub-domains in the foundation in the "High-Quality Health System" framework suggested by the Lancet Global Health Commission: population and their health needs, governance of the health sector, platforms for health care delivery, workforce, and tools such as medicine and equipment (Figure 1) (Kruk et al., 2018).

Empowered women raise a voice for better service to providers, facilities, or to government (Lewis, Ndiaye, Manzi, & Kruk, 2022). In more densely populated areas, information sharing will more actively occur, and hence, people's feedback on service quality would be more immediate. Previous studies have shown that high level of ethnic diversity is associated with poor governance, especially in terms of public good provision. In ethnically-heterogenous communities, the perceived value of a particular health service relative to other services is likely to differ due to cultural variation. Additionally, conflict between ethnic groups is frequent because they represent the interests of their own ethnic group (Habyarimana, Humphreys, Posner, & Weinstein, 2007). Ethnic diversity, on the other hand, may offers advantages as well. Inter-group contact makes it more difficult to uphold socially conservative social norms,

including those regarding the subservient role of women (Figure 1) (Dinku, Fielding, & Genç, 2019; Fielding & Lepine, 2017).

Large facilities, including hospitals and large health centers, may offer technical support to lower-level facilities. Private facilities might trigger more competition among facilities for better care (Zelder, 2000). The composition of the workforce in the region can impact the service quality through spillover effects from better-performing providers to others by sharing knowledge and experience among peers (Wong, McNamara, & Greenberg, 2004). Specifically, medical doctors (MD) (or quasi-MD) can teach other less-qualified health workers. A high proportion of top-performing providers in the region means that there are many exemplary providers who other providers can benchmark. Better tools allow providers to be able to perform more diagnostic tests or treatments (Figure 1).

eAppendix2. SPA dataset

SPA consists of four distinct modules; facility audits to collect data on available infrastructure, health provider interviews, direct observation of protocols for different types of care (ex. antenatal care, sick childcare, or family planning) to assess process quality, and finally, an exit interview with caregivers to assess their satisfaction with the service and examine demographic characteristics. For process quality for sick childcare, patients presenting for sick childcare were sampled within each sampled health facility, and consultations for them were observed and assessed by trained observers.

Table S1. Recommended tasks in Integrated Management of Childhood Illnesses (IMCI) used to construct the technical quality score of sick childcare

For children older than 2 months	For children 2 months or younger
· Provider asked / caretaker mentioned<br>- if child was unable to drink or breastfeed<br>- cough or difficult breathing<br>- diarrhea<br>- fever<br>- about vomiting<br>- convulsions<br>- ear pain<br>· Provider asked<br>- about mother's HIV* status<br>- if child received vitamin A within past 6 months<br>- if child received any deworming medication in last 6 mon.<br>· Provider checked<br>- palms / conjunctiva / mouth for pallor<br>- for oedema<br>- vaccination card or vaccinated<br>· Provider counted respiration for 60 seconds<br>· Provider weighed client<br>· Provider plotted weight on growth chart<br>· Provider took temperature<br>· Provider explained dosing if medication prescribed<br>· Provider recommended food / liquid intake<br>· Provider described ≥1 danger sign requiring return to facility<br>· Provider discussed follow-up appointment<br>· Provider stated diagnosis to caretaker	· Provider asked / caretaker mentioned<br>- if child was unable to drink or breastfeed<br>- diarrhea<br>- convulsions<br>· Provider asked<br>- about normal (breast)feeding pattern<br>- about (breast)feeding pattern during this illness<br>- about mother's HIV* status<br>- if child received vitamin A within past 6 months<br>· Provider counted respiration for 60 seconds<br>· Provider weighed client<br>· Provider plotted weight on growth chart<br>· Provider took temperature<br>· Provider looked into child's mouth<br>· Provider checked vaccination card or vaccinated<br>· Provider explained dosing if medication prescribed<br>· Provider recommended food / liquid intake<br>· Provider described ≥1 danger sign requiring return to facility<br>· Provider discussed follow-up appt (sick child)

*: Human Immunodeficiency Virus

Table S2. Detailed information on the region in each country

Country	Administrative levels
Senegal	Senegal is composed of 14 regions, 45 departments, and 103 arrondissements.[1] The 14 regions are administered by a *Conseil Régionaux*, which is elected by population weight at the arrondissement level. Department has no independent political and arrondissement is purely administrative structure.[2]
Afghanistan	Afghanistan is divided into 34 provinces, which are the primary administrative divisions[3]. SPA* was surveyed only in 7 provinces (balkh, Hirat, Kabul, Kandahar, Kunduz, Nagarhar, and Paktya).
Nepal	Nepal is divided into five development regions, 14 administrative zones and 75 districts prior to the promulgation of a new constitution in 2015. Developmental region was creased to promote national unity by removing regional imbalances and also by utilizing the natural resources of the mountains and hills in a proportional manner. All developmental regions are under direction and supervision of their regional headquarters.[4]
DR Congo	DR Congois divided into the capital city of Kinshasa and 25 provinces, and 145 territories. Provinces have both an executive body with a leader and a deliberative body which was to elect the leader.[5]

*SPA: Service Provision Assessment

[1] The World Factbook. Washington, DC: Central Intelligence Agency (Retrieved from: https://www.cia.gov/library/publications/the-world-factbook/index.html); 2018.
[2] Wikipedia. Regions of Senegal (https://en.wikipedia.org/wiki/Regions_of_Senegal) (Accessed on Sep 14 2022)
[3] Wikipedia. Provinces of Afghanistan (https://en.wikipedia.org/wiki/Provinces_of_Afghanistan) Accessed Sep 14, 2022.
[4] Wikipedia. Development regions of Nepal (https://en.wikipedia.org/wiki/Development_regions_of_Nepal) (Accessed on Sep 14 2022).
[5] Wikipedia. Subdivisions of the Democratic Republic of the Congo (https://en.wikipedia.org/wiki/Subdivisions_of_the_Democratic_Republic_of_the_Congo) (Accessed on Sep 14 2022)

Table S3. Population density in regions of each country

Region	Pop density (/km²)
**DR Congo**	
Sud-kivu	89
Kinshasa	950
Kongo central	84
Mai-ndombe	14
Kwilu	66
Kwango	22
Equateur	16
Sud ubangi	53
Nord ubangi	26
Mongala	31
Tshuapa	10
Tshopo	13
Bas-uele	7
Haut uele	21
Ituri	65
Nord-kivu	97
Haut-katanga	30
Lualaba	14
Ht lomami	23
Tanganika	22
Kasaï-oriental	570
Sankuru	13
Lomami	36
Kasaï	33
Kasaï-central	50
Maniema	15

Region	Pop density (/km²)
**Afghanistan**	
Balkh	93
Hirat	38
Kabul	1,151
Kandahar	26
Kunduz	141
Nagarhar	223
Paktya	110
**Nepal**	
Eastern development region	204
Central development region	352
Western development region	168
Mid-western development region	84
Far-western development region	131
**Senegal**	
Dakar	6464
Diourbel	348
Fatick	115
Kaffrine	59
Kaokack	206
Kedougou	10
Kolda	55
Louga	38
Matam	23
Saint louis	53
Sediou	70
Tambacounda	18
Thies	302
Ziguinchor	84

Table S4. Missing rate at each level overall and by country

	Level	Afghanistan 2018	DR Congo 2018	Nepal 2015	Senegal 2019	Total
Original sample (N)	Individual	576	2,673	2,229	885	6,363
	Provider	133	1,303	860	255	2,551
	Facility	84	992	673	253	2,002
Final analytic sample (N)	Individual	467	2,425	2,072	813	5,777
	Provider	113	1,230	826	238	2,407
	Facility	73	946	657	237	1,913
Missing rate (%)	Individual	18.9	9.3	7	8.1	9.2
	Provider	15	5.6	4	6.7	5.6
	Facility	13.1	4.6	2.4	6.3	4.4

Table S5. Comparison of original sample and analytical sample by country

		Afghanistan 2018		DR Congo 2018		Nepal 2015		Senegal 2019	
Variables	Categories	Original (%)	Final (%)	Original (%)	Final (%)	Original (%)	Final (%)	Original (%)	Final (%)
Individual variables									
Caregiver's age (years)	≦25	30.7	29.8	35.0	35.0	54.0	54.2	40.7	40.7
	25-40	57.0	57.6	55.9	56.1	40.1	39.7	50.2	50.3
	>40	12.3	12.6	9.2	8.9	5.9	6.0	9.1	9.0
Caregiver's education level	< secondary graduate	66.9	63.4	44.7	42.0	42.2	41.4	79.6	79.2
	≧ secondary graduate	33.1	36.6	55.3	58.0	57.8	58.6	20.4	20.8
Relation to child	mother	66.3	65.5	78.1	78.6	80.6	82.1	85.2	85.1
	father	21.0	21.6	13.3	13.5	9.1	9.3	5.8	5.7
	sibling/grandparents/others	12.7	12.8	8.6	7.9	10.3	8.5	9.0	9.2
Provider variables									
Gender	male	98.5	98.2	84.1	83.7	75.5	75.4	56.5	55.9
	female	1.5	1.8	15.9	16.3	24.5	24.6	43.5	44.1
Qualification	MD or quasi-MD*	100	100	46.4	46.7	32.8	32.9	9.8	8.8
	Others	0.0	0.0	53.6	53.3	67.2	67.1	90.2	91.2
Having job description	no	27.7	29.2	51.6	51.9	73.1	73.0	48.4	47.9
	yes	72.3	70.8	48.4	48.1	26.9	27.0	51.6	52.1
Recent training for sick childcare	no	93.8	95.6	78.7	78.5	86.8	86.8	58.7	59.2
	yes	6.2	4.4	21.3	21.5	13.2	13.2	41.3	40.8
Facility variables									
Facility level	hospital	67.9	71.2	47.8	47.1	26.3	25.7	8.3	7.6
	large health center	0.0	0.0	16.4	16.7	0.0	0.0	0.0	0.0
	health center	32.1	28.8	35.8	36.2	29.7	29.8	91.7	92.4
	health post +others	0.0	0.0	0.0	0.0	44.0	44.4	0.0	0.0
Facility type	public	17.9	17.8	62.6	62.1	88.0	88.7	87.7	90.3
	private for profit	66.7	69.9	9.2	9.5	10.1	9.3	4.3	3.4
	private not for profit	15.5	12.3	28.2	28.4	1.9	2.0	7.9	6.3
Patient load per providers	≤2	29.6	27.4	93.0	93.0	81.1	81.0	66.9	66.7
	>2 & ≤3	17.3	17.8	3.7	3.7	7.9	8.1	13.2	13.5
	>3	53.1	54.8	3.3	3.3	11.0	11.0	19.8	19.8
Guideline for IMCI** observed	no	86.9	86.3	49.3	49.3	52.1	51.8	13.9	12.2
	yes	13.1	13.7	50.7	50.7	47.9	48.2	86.1	87.8

*MD: Medical Doctor/**IMCI: Integrated management of childhood illness

Table S6. Descriptive statistics of analytic sample by country

		Afghanistan 2018	DR Congo 2018	Nepal 2015	Senegal 2017
**Individual variables**	**Total**	467	**2425**	**2072**	**813**
Caregiver's age (years)	≦25	139 (29.8%)	848 (35.0%)	1124 (54.2%)	331 (40.7%)
	25-40	269 (57.6%)	1360 (56.1%)	823 (39.7%)	409 (50.3%)
	>40	59 (12.6%)	217 (8.9%)	125 (6.0%)	73 (9.0%)
Caregiver's education level	< secondary graduate	296 (63.4%)	1019 (42.0%)	858 (41.4%)	644 (79.2%)
	≧ secondary graduate	171 (36.6%)	1406 (58.0%)	1214 (58.6%)	169 (20.8%)
Relation to child	mother	306 (65.5%)	1907 (78.6%)	1702 (82.1%)	692 (85.1%)
	father	101 (21.6%)	327 (13.5%)	193 (9.3%)	46 (5.7%)
	sibling/grandparents/others	60 (12.8%)	191 (7.9%)	177 (8.5%)	75 (9.2%)
**Provider variables**	**Total**	113	1230	826	238
Gender	male	111 (98.2%)	1030 (83.7%)	623 (75.4%)	133 (55.9%)
	female	2 (1.8%)	200 (16.3%)	203 (24.6%)	105 (44.1%)
Qualification	MD and quasi-MD†	113 (100.0%)	575 (46.7%)	272 (32.9%)	21 (8.8%)
	others	0 (0.0%)	655 (53.3%)	554 (67.1%)	217 (91.2%)
Having job description	no	33 (29.2%)	638 (51.9%)	603 (73.0%)	114 (47.9%)
	yes	80 (70.8%)	592 (48.1%)	223 (27.0%)	124 (52.1%)
Having received recent training for sick childcare	no	108 (95.6%)	965 (78.5%)	717 (86.8%)	141 (59.2%)
	yes	5 (4.4%)	265 (21.5%)	109 (13.2%)	97 (40.8%)
**Facility variables**	**Total**	73	946	657	237
Facility level	hospital	52 (71.2%)	446 (47.1%)	169 (25.7%)	18 (7.6%)
	large health center	0 (0.0%)	158 (16.7%)	0 (0.0%)	0 (0.0%)
	health center	21 (28.8%)	342 (36.2%)	196 (29.8%)	219 (92.4%)
	health post + others	0 (0.0%)	0 (0.0%)	292 (44.4%)	0 (0.0%)
Facility type	public	13 (17.8%)	587 (62.1%)	583 (88.7%)	214 (90.3%)
	private for profit	51 (69.9%)	90 (9.5%)	61 (9.3%)	8 (3.4%)
	private not for profit	9 (12.3%)	269 (28.4%)	13 (2.0%)	15 (6.3%)
Patient load per day per provider	≤2	20 (27.4%)	880 (93.0%)	532 (81.0%)	158 (66.7%)
	>2 & ≤3	13 (17.8%)	35 (3.7%)	53 (8.1%)	32 (13.5%)
	>3	40 (54.8%)	31 (3.3%)	72 (11.0%)	47 (19.8%)
Guideline for IMCI‡ observed	no	63 (86.3%)	466 (49.3%)	340 (51.8%)	29 (12.2%)
	yes	10 (13.7%)	480 (50.7%)	317 (48.2%)	208 (87.8%)

Continuous variables																
	Afghanistan 2018				DR Congo 2018				Nepal 2015				Senegal 2017			
**Facility variables**	Mean	SD	Min	Max	Mean	SD	Min	Max	Mean	SD	Min	Max	Mean	SD	Min	Max
Service readiness score for sick childcare	0.57	0.04	0.53	0.73	0.67	0.08	0.46	0.81	0.58	0.03	0.56	0.65	0.67	0.05	0.53	0.77
**Regional variables**																
Proportion of top 5th in women's empowerment	0.05	0.04	0.01	0.11	0.24	0.12	0.03	0.49	0.30	0.08	0.19	0.38	0.12	0.09	0.02	0.29
Population density (1,000 per km^2)	0.06	0.53	0.03	1.15	0.09	0.21	0.01	0.95	0.22	0.10	0.08	0.35	1.23	2.40	0.01	6.46
Ethnic diversity	0.48	0.25	0.14	0.75	0.23	0.24	0.01	0.75	0.78	0.07	0.66	0.84	0.57	0.16	0.27	0.75
Proportion of large facilities	0.51	0.25	0.23	0.93	0.22	0.15	0.06	0.80	0.08	0.02	0.06	0.10	0.03	0.03	0.00	0.09
Proportion of private facilities	0.84	0.10	0.67	0.95	0.32	0.23	0.03	0.82	0.05	0.02	0.02	0.07	0.11	0.11	0.00	0.43
Proportion of MD or quasi-MD†	1.00	0.00	1.00	1.00	0.22	0.23	0.00	0.69	0.12	0.05	0.05	0.19	0.04	0.05	0.00	0.13
Proportion of providers in top 5th in quality score	0.04	0.07	0.00	0.17	0.22	0.12	0.04	0.47	0.18	0.04	0.12	0.22	0.32	0.29	0.00	0.90
Proportion of providers with recent IMCI‡ training	0.00	0.00	0.00	0.00	0.31	0.47	0.00	1.00	0.40	0.55	0.00	1.00	0.36	0.50	0.00	1.00
Proportion of providers with job description	0.78	0.23	0.32	1.00	0.45	0.23	0.07	0.92	0.29	0.08	0.18	0.39	0.53	0.32	0.03	1.00
Service readiness score for sick childcare.	0.70	0.05	0.65	0.76	0.62	0.08	0.43	0.77	0.68	0.04	0.65	0.74	0.72	0.05	0.65	0.80

†MD: Medical Doctor / ‡IMCI: Integrated management of childhood illness

Table S7. Bivariate association with process quality score for sick childcare

Variables	b	P value	95% CI
**Client characteristics**			
Caretaker's age (ref: ≤ 25)			
25-40	0.75	0.046	(0.01, 1.49)
>41	-1.09	0.112	(-2.44, 0.26)
Caretaker's education level (ref: < secondary)			
≥ secondary graduate	-0.34	0.342	(-1.05, 0.36)
Relation to child (ref: mother)			
Father	-2.09	0	(-3.20, -0.98)
Sibling/grandparents/others	-1.85	0.004	(-3.10, -0.59)
Total number of child symptoms	2.00	0	(1.75, 2.25)
**Provider characteristics**			
Sex (Ref: male)			
Female	0.33	0.458	(-0.54, 1.19)
MD or quasi-MD (ref: no)			
Yes	-2.19	0	(-2.90, -1.48)
Having job description (ref: no)			
Yes	2.66	0	(1.95, 3.37)
Received recent training for sick childcare (ref: no)			
Yes	3.94	0	(3.07 4.81)
**Facility characteristics**			
Facility level (ref: hospital)			
Large health centers	4.08	0	(2.65, 5.61)
Health centers	2.26	0	(1.48, 3.04)
Health post and others	-1.75	0.003	(-2.89, -0.61)
Type (ref: public)			
Private for profit	-0.35	0.521	(-1.43, 0.72)
Private not for profit	1.09	0.03	(0.11, 2.08)
Patients case load (ref: ≤2)			
>2 & ≤3	-3.02	0	(-4.14, -1.89)
>3	-4.85	0	(-5.76, -3.94)
Guideline for IMCI observed (ref: no)			
Yes	4.09	0	(3.39, 4.79)
Service readiness for sick childcare (0~1)	35.33	0	(30.75, 39.90)
**Region characteristics**			
Proportion of top 5th quintiles in women empowerment	-0.99	0.511	(-3.95, 1.97)
Population density (1,000 per km^2)	0.30	0.09	(-0.05, 0.65)
Ethnic diversity	-7.36	0	(-8.48, -6.23)
Proportion of large facilities	-0.74	0.53	(-3.04, 1.57)
Proportion of private facilities	-1.48	0.026	(-2.78, -0.18)

Proportion of MD or quasi-MD	-4.77	0	(-6.03, -3.52)
Proportion of providers in top 5th in quality score	33.37	0	(30.96, 35.77)
Proportion of providers who received recent IMCI training	17.6	0.0	(15.26, 19.93)
Proportion of providers with job description	6.28	0	(4.83, 7.72)
Service readiness score for sick childcare.	-2.95	0.235	(-7.83, 1.92)

Table S8. Results on the cross-level interactions.

	M4-1		M4-2		M4-3		M4-4	
	b	P value	b	P value	b	P value	b	P value
**Provider characteristics**								
Gender (ref: male)								
Female	0.23	0.826						
MD or quasi-MD (ref: no)								
Yes			3.8	0.001				
Having job description (ref: no)								
Yes					-1.05	0.26		
Having received recent IMCI training (ref=no)								
Yes							2.92	0.006
**Region characteristics**								
Proportion of providers in top 5th in quality score	30.27	0	34.3	0	21.30	0	31.42	0
**Interaction term**								
Female x Prop. providers in top 5th in quality score	-0.45	0.916						
MD (or quasi-MD) x Prop. in top 5th in quality score			-16.6	0				
Job description x Prop. providers in top 5th in quality score					12.01	0.002		
Recent IMCI training x Prop. providers in top 5th in quality score							-5.33	0.191

	M4-5		M4-6		M4-7		M4-8	
	b	P value	b	P value	b	P value	b	P value
**Provider characteristics**								
Gender (ref: male)								
Female	0.37	0.679						
MD or quasi-MD (ref: no)								
Yes			-0.90	0.366				
Having job description (ref: no)								
Yes					1.78	0.013		
Having received recent IMCI training (ref=no)								
Yes							2.95	0.001
**Region characteristics**								
Prop. large facilities	8.26	0.004	2.55	0.536	9.64	0.004	9.65	0.001
**Interaction term**								
Female x Prop. large facilities	-1.70	0.736						
MD (or quasi-MD) x Prop. large facilities			8.00	0.064				
Job description x Prop. large facilities					-2.75	0.4		
Recent IMCI training x Prop. large facilities							-7.81	0.068

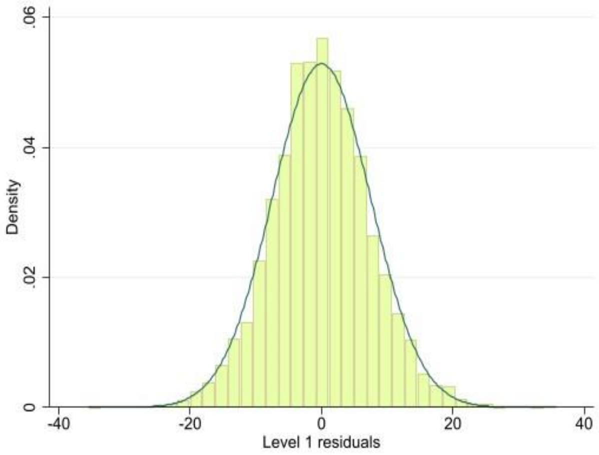

Skewness: 0.1220
Kurtosis: 3.5924(excess kurtosis:0.5924)

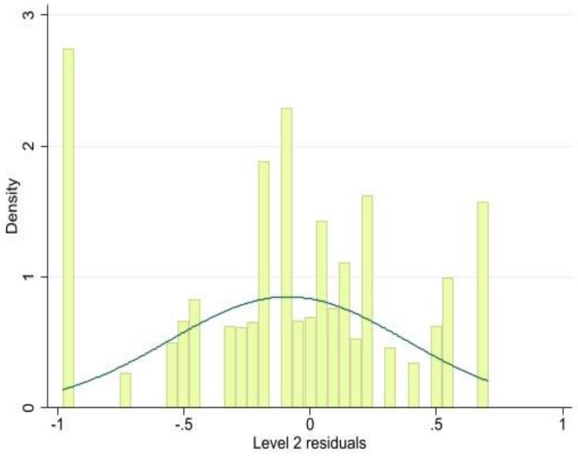

Skewness: -0.3713
Kurtosis: 2.6038(excess kurtosis:-0.3962)

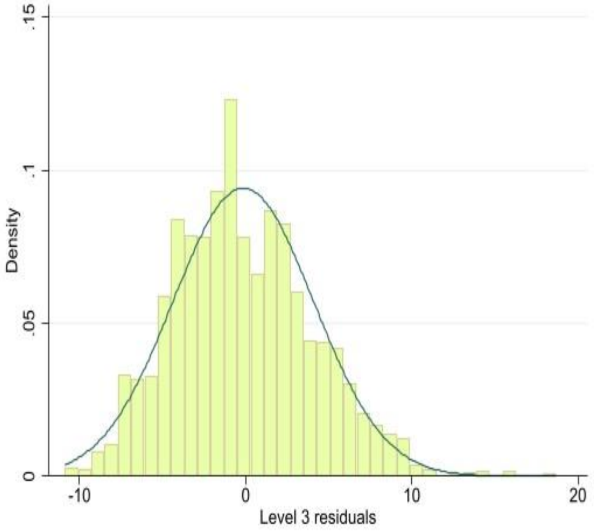

Skewness: 0 .4185
Kurtosis: 3.3007 (excess kurtosis: 0.3007)

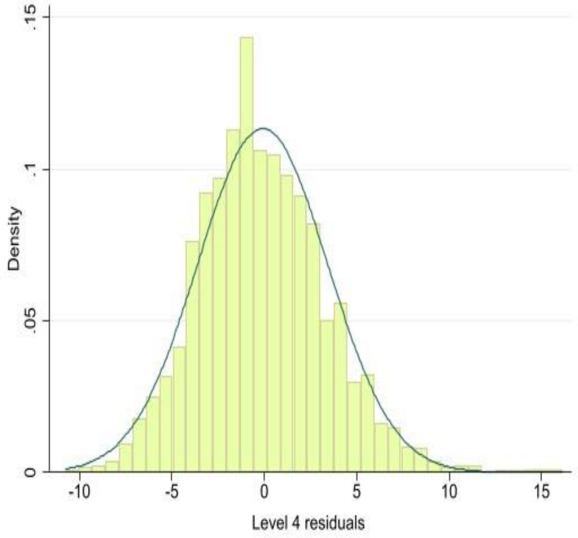

Skewness: 0.4868
Kurtosis: 3.9090 ((excess kurtosis:0.9090)

Figure S1. Normality check of residuals at level 1, 2, 3, and 4

References for eAppendix1.

Barber, S. L., Bertozzi, S. M., & Gertler, P. J. (2007). Variations In Prenatal Care Quality For The Rural Poor In Mexico. *Health Affairs, 26*(Supplement 2), w310-w323. doi:10.1377/hlthaff.26.3.w310

Dinku, Y., Fielding, D., & Genç, M. (2019). Neighbourhood ethnic diversity, child health outcomes and women's empowerment. *The Journal of Development Studies, 55*(9), 1909-1927.

Fielding, D., & Lepine, A. (2017). Women's empowerment and wellbeing: evidence from Africa. *The journal of development studies, 53*(6), 826-840.

Habyarimana, J., Humphreys, M., Posner, D. N., & Weinstein, J. M. (2007). Why does ethnic diversity undermine public goods provision? *American political science review, 101*(4), 709-725.

Hansen, P. M., Peters, D. H., Edward, A., Gupta, S., Arur, A., Niayesh, H., & Burnham, G. (2008). Determinants of primary care service quality in Afghanistan. *International Journal for Quality in Health Care, 20*(6), 375-383. doi:10.1093/intqhc/mzn039 %J International Journal for Quality in Health Care

Kruk, M. E., Gage, A. D., Arsenault, C., Jordan, K., Leslie, H. H., Roder-DeWan, S., . . . Doubova, S. V. (2018). High-quality health systems in the Sustainable Development Goals era: time for a revolution. *The Lancet global health, 6*(11), e1196-e1252.

Leslie, H. H., Gage, A., Nsona, H., Hirschhorn, L. R., & Kruk, M. E. (2016). Training and supervision did not meaningfully improve quality of care for pregnant women or sick children in sub-Saharan Africa. *Health Affairs, 35*(9), 1716-1724.

Lewis, T. P., Ndiaye, Y., Manzi, F., & Kruk, M. E. (2022). Associations between women's empowerment, care seeking, and quality of malaria care for children: A cross-sectional analysis of demographic and health surveys in 16 sub-Saharan African countries. *Journal of global health, 12*.

Pariyo, G. W., Gouws, E., Bryce, J., & Burnham, G. (2005). Improving facility-based care for sick children in Uganda: training is not enough. *Health policy planning, 20*(suppl_1), i58-i68.

Sato, M., Maufi, D., Mwingira, U. J., Leshabari, M. T., Ohnishi, M., & Honda, S. (2017). Measuring three aspects of motivation among health workers at primary level health facilities in rural Tanzania. *PloS one, 12*(5), e0176973.

Soeters, R., Habineza, C., & Peerenboom, P. B. (2006). Performance-based financing and changing the district health system: experience from Rwanda. *Bulletin of the World Health Organization, 84*(11), 884-889.

Wong, H. S., McNamara, P., & Greenberg, W. (2004). Provider competition and health care quality: challenges and opportunities for research. *International Journal of Health Care Finance Economics, 4*, 99-111.

Zelder, M. (2000). How Private Hospital competition can improve Canadian health care. *Retrieved from https://policycommons.net/artifacts/419835/how-private-hospital-competition-can-improve-canadian-health-care/1390375/ on 04 Mar 2023. CID: 20.500.12592/44mdjp.*
